# Ultrasonic versus conventional gap arthroplasty for the release of ankylosis of temporomandibular joint: a prospective cohort study

**DOI:** 10.1038/s41598-018-36955-3

**Published:** 2019-01-23

**Authors:** Tingting Jia, Li Wang, Youbai Chen, Rui Zhao, Liang Zhu, Lejun Xing, Naman Rao, Jie Zhang, Qixu Zhang, Meredith August, Yan Han, Haizhong Zhang

**Affiliations:** 1 Department of Oral and Maxillofacial Surgery, Chinese PLA General Hospital, Beijing, 100853 China; 2 Department of Plastic Surgery, Chinese PLA General Hospital, Beijing, 100853 China; 3 Harvard Medical School, Boston, MA 02115 USA; 4 Department of Oral and Maxillofacial Surgery, Massachusetts General Hospital, Boston, MA 02114 USA; 5 Department of Plastic Surgery, The University of Texas, MD Anderson Cancer Center, Houston, TX 77030 USA

## Abstract

The purpose of this study was to compare the clinical outcomes of ultrasonic surgery to the conventional bone cutting technique using bur and saw for the release of ankylosis of temporomandibular joint. We conducted a prospective cohort study on 25 patients with 38 ankylotic joints at Chinese PLA General Hospital from March 01, 2012 to March 01, 2016. Patients were followed up at least 2 years postoperatively. The primary outcome was the intraoperative blood loss per joint. The secondary outcome was the long-term (≥2 years) improvement of maximum mouth opening. The blood loss was significantly reduced in the ultrasonic group compared to the conventional group (107.3 ± 62.3 ml vs. 186.3 ± 92.6 ml, P = 0.019). The long-term improvements of maximum mouth opening were substantial and stable in both groups (33.5 ± 4.8 mm in the ultrasonic group vs. 29.2 ± 6 mm in the conventional group, P = 0.06). Multivariate linear regression analysis showed a significant association between blood loss and technique used (coefficient: 66.3, 95% confidence interval: 22.1,110.4, P = 0.006). The ultrasonic surgery was associated with less intraoperative blood loss when compared to the conventional method for the release of ankylosis of temporomandibular joint while providing a stable and comparable long-term improvement of maximum mouth opening.

## Introduction

Ankylosis of temporomandibular joint (ATMJ) is characterized by fusion and immobility between the mandibular condyle and glenoid fossa, leading to a progressive limitation of mouth opening^1^. ATMJ is commonly caused by trauma, infection, rheumatologic disease, and previous surgery^2^. In addition to profound functional concerns including mastication, digestion, speech, and oral hygiene, ATMJ can result in facial asymmetric growth and resistant retrognathia^3^.

Various surgical options including gap, interpositional, and reconstructive arthroplasty have been introduced to correct the ATMJ. Gap arthroplasty using conventional rotary bur and saw is one of the most commonly performed procedures to remove the ankylotic mass and restore the space between the condyle and glenoid fossa, providing good mouth opening^4^. However, resection of the ankylotic block using conventional bone cutting technique is challenging due to the complexity of surrounding nerves and vessels, which may cause nerve injury and bleeding, thus increasing the risk of hematoma, infection, scarring, excessive bone regeneration, and reankylosis^5^.

Ultrasonic surgery is a precise, safe, and minimally invasive technique for selective bone cutting by employing ultrasonic frequencies^6^. Since its first introduction, it has been widely used in various fields including orthopedics, neurosurgery, otorhinolaryngology, plastic surgery, dentistry and craniomaxillofacial surgery^7–12^. However, few studies have evaluated its use for release of ATMJ. The purpose of this prospective cohort study is to answer the following clinical question: Among patients with ATMJ, does ultrasonic surgery reduce the intraoperative blood loss when compared to the conventional bone cutting technique using rotary instruments?

## Results

### Participants

A total of 25 participants including 13 females and 12 males, aged in average 34.1 ± 16.8 years, with 38 ankylotic joints (12 unilateral and 13 bilateral), were enrolled in this study. The number of patients who presented with type II, type III, and type IV ankylosis was 15(60%), 6(24%), and 4(16%), respectively. Trauma was the leading cause of ATMJ (18 patients, 72%), following by unknown etiology (3 patients, 12%), infectious diseases (2 patients of osteomyelitis, 8%) and rheumatologic diseases (2 patients, 1 ankylosing spondylitis and 1 rheumatoid arthritis, 8%). The operation duration per joint was 2.1 ± 0.8 hours in the ultrasonic group and 2.1 ± 1.1 hours in the conventional group. The median of follow-up was 32 months, ranged from 24 to 72 months. There was no statistically significant difference of these variables between the two groups **(**Table 1).

**Table 1:** Demographics, ankylosis characteristics, operation duration and follow-up time.

	Total (n = 25)	Ultrasonic (n = 13)	Conventional (n = 12)	P-value
Age, mean ± SD, years	34.1 ± 16.8	34.8 ± 17	33.4 ± 17.4	0.85
Gender,male(%)	12(48%)	7(54%)	5(42%)	0.7
Number of ankylosis	38	20	18	
Affected side, bilateral(%)	13(52%)	7(54%)	6(50%)	0.84
Type				
II(%)	15(60%)	7(54%)	8(66%)	0.54
III(%)	6(24%)	4(31%)	2(17%)	0.42
IV(%)	4(16%)	2(15%)	2(17%)	0.89
Etiology				
Traumatic(%)	18(72%)	10(77%)	8(67%)	0.58
Infectious(%)	2(8%)	1(8%)	1(8%)	1
Rheumatic(%)	2(8%)	2(15%)	0	0.16
Other(%)	3(12%)	0	5(25%)	0.06
Operation duration per side, hours	2.2 ± 0.9	2.1 ± 0.8	2.1 ± 1.1	0.94
Follow-up, median(min,max),months	32(24, 72)	27(24,72)	34(24,60)	0.46

### Blood loss and other complications

We observed a significant reduction of intraoperative blood loss per joint in the ultrasonic group compared to the conventional group (107.3 ± 62.3 ml vs. 186.3 ± 92.6 ml, P = 0.019). Although the drainage duration in the ultrasonic group (4.3 ± 1.3 days) was shorter than the conventional group (5.6 ± 1.8 days), the difference was not statistically significant. One patient in the conventional group developed local infection 6 days after surgery, which was completely resolved after 1 week of antibiotic treatment. No hematoma or facial nerve injury occurred in either group. No patients required a blood transfusion during or after the surgery. The average pain visual analogue scale was 3.3 ± 0.9 in the ultrasonic group and 3.8 ± 1.1 in the conventional group (P = 0.28).

### Long-term improvement of MMO

The preoperative, intraoperative, and 2-year postoperative MMO was shown in Table 2. Although the long-term improvements of MMO were not statistically significant, they were substantial and stable in both groups (33.5 ± 4.8 mm in the ultrasonic group and 29.2 ± 6 mm in the conventional group, P = 0.06). The relapse of MMO in the ultrasonic group was 1.6 ± 2.9 mm and 4.7 ± 4.8 mm in the conventional group (P = 0.66). No re-ankylosis occurred in either group **(**Table 2**)**.

**Table 2:** Complications and changes of maximum mouth opening.

	Total (n = 25)	Ultrasonic (n = 13)	Conventional (n = 12)	P-value
Complications				
Blood loss per joint, mean ± SD, ml	145.2 ± 86. 6	107.3 ± 62.3	186.3 ± 92.6	0.019*
Drainage duration, days	4.9 ± 1.7	4.3 ± 1.3	5.6 ± 1.8	0.052
Infection (%)	1 (4%)	0	1 (8.3%)	0.29
Pain VAS, mean ± SD	3.5 ± 1	3.3 ± 0.9	3.8 ± 1.1	0.28
MMO, mean ± SD, mm				
Preoperative(t0)	4.3 ± 4.1	3.3 ± 3.4	5.3 ± 4.7	0.23
Intraoperative (t1)	37 ± 2.1	36.8 ± 2.1	37.3 ± 2.2	0.16
>2 year postoperative(t2)	35.3 ± 5.14	36.9 ± 4.6	33.6 ± 5.4	0.11
Improvement(t2–t0)	31.4 ± 5.8	33.5 ± 4.8	29.2 ± 6	0.06
Relapse(t2–t1)	3.1 ± 4.2	1.6 ± 2.9	4.7 ± 4.8	0.66

### Correlation and Regression

Pearson correlation analysis showed a positive correlation between blood loss and age, type of ATMJ, technique, preoperative MMO, and operation duration. Univariate linear regression showed only technique and operation duration had a p-value less than 0.05 (0.019 for technique and < 0.001 for operation duration). The output of LASSO regression showed the coefficient of technique and operation duration was 45.7 and 64.8, respectively. Multiple linear regression analysis showed significant association between blood loss and technique (coefficient: 66.3, 95% confidence interval: 22.1,110.4, P = 0.006), and blood loss and operation duration (coefficient: 66.7, 95% confidence interval: 43, 90.5, P < 0.001). A comparison of the effect strength based on the standardized coefficients demonstrated the effect of operation duration (0.72) and technique (0.39) on blood loss was greater than other variables.

## Discussion

The results of the present study indicated that gap arthroplasty using ultrasonic bone cutting technique significantly reduced the intraoperative bleeding when compared to the conventional technique while providing stable and comparable long-term improvement of MMO at a minimum 2-year follow-up.

Although various surgical procedures including the gap, interpositional, and reconstructive arthroplasty have been described to correct ATMJ in the literature^13^, there is no consensus on the best treatment method. Some studies showed that the long-term mouth opening and recurrence rate after interpositional and reconstructive arthroplasty were superior to the gap arthroplasty^14–16^, whereas other researches demonstrated a better or comparable clinical outcome of gap arthroplasty^17,18^. We found a substantial improvement of MMO (average 31.4 ± 5.8 mm) and stable long-term postoperative MMO (average 35.3 ± 5.1 mm) without reankylosis in our cohorts regardless of the applied bone cutting technique, indicating gap arthroplasty is a predictable procedure for the treatment of ATMJ.

In accordance with previous studies, trauma was the most common etiology of ATMJ^19^. It was noteworthy that a considerable proportion of patients had a history of chin injury due to accidental fall during their childhood, which was initially asymptomatic but gradually presented a limitation of mouth opening. In our clinical practice, the laceration of the chin due to falling is one of the most common traumas among pediatric patients in our emergency service. It is essential to pay more attention and close follow-up to the pediatric patient who experienced a chin trauma.

The complex anatomy involving plenty of blood vessels and nerves around TMJ increases the risk of bleeding and nerve injury during resection of ankylotic block^20^. Bleeding may cause hematoma, scarring, thus resulting in excessive bone regeneration and reankylosis. Intraoperative hemostasis is a serious challenge due to the limited operative space and exposure. To protect the blood vessels and nerves, an extended incision, wide exposure of the ankylotic mass, aggressive retraction, sacrifice of soft tissue, and a protective shield are often required in conventional arthroplasty, which may lead to a prolonged operation duration, postoperative swelling, pain, and scar^21^. A major advantage of ultrasonic surgery is the precise and selective cutting for mineralized structures, thus sparing soft tissue and significantly reducing blood loss^22^. The present study showed a significant reduction in blood loss per joint in the ultrasonic group compared to the conventional group. Correlation and regression analysis also demonstrated that ultrasonic bone cutting was significantly associated with less intraoperative blood loss. We would expect a reduction of 66.3 ml of blood loss by applying ultrasonic osteotomy compared to the conventional technique, holding other variables constant. Furthermore, ultrasonic surgery provided a clean osteotomy field due to the cavitation effect of ultrasonic vibration^23–25^. The concomitant irrigation system flushed out the bony debris, minimizing the risk of heterotopic bone formation and reankylosis caused by the residual bony debris.

**Figure 1:**
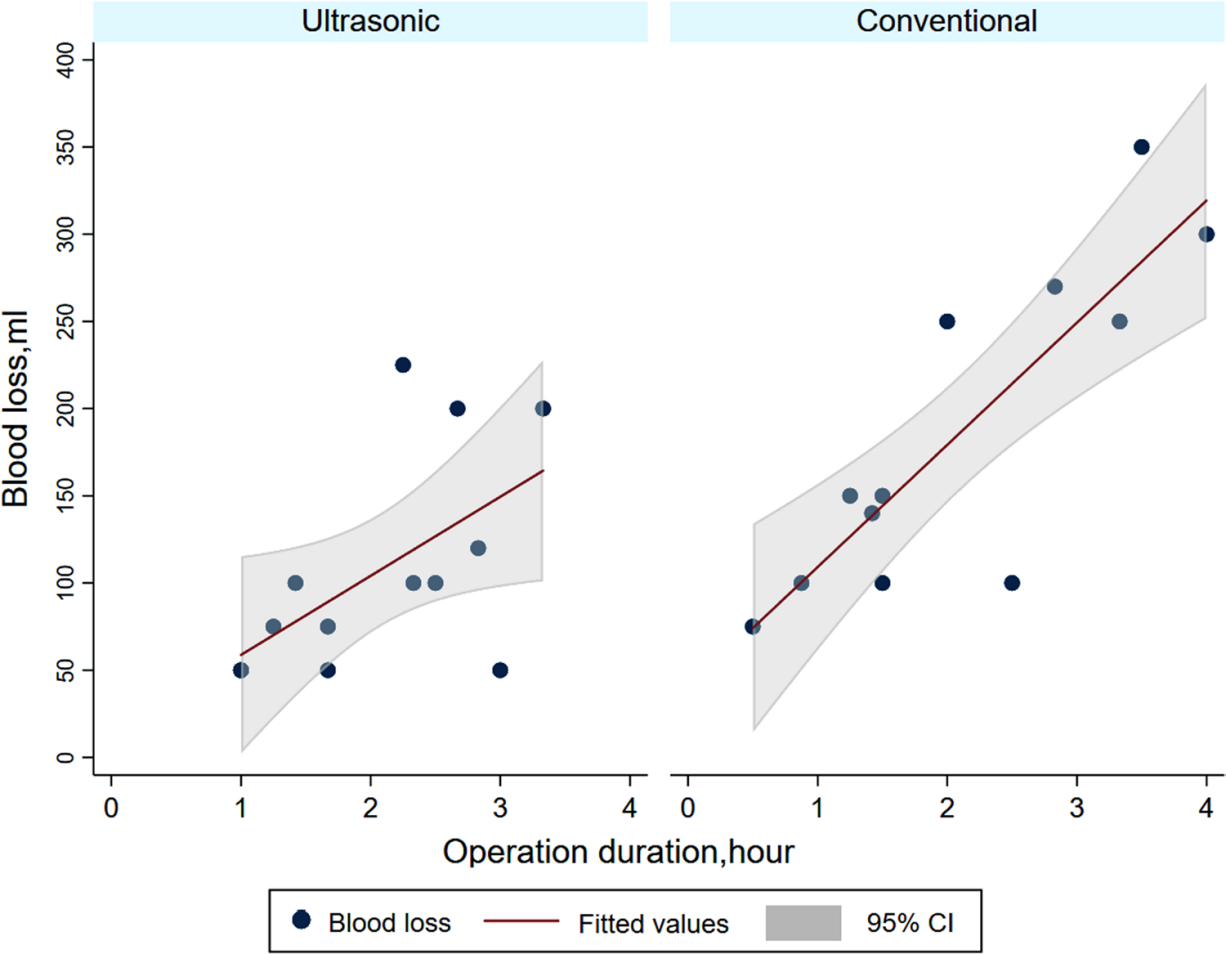
Linear fit of blood loss and operation duration by different bone cutting techniques. Intergroup comparison of these models further confirmed similar positive linear associations between blood loss and operation duration regardless of the technique, except the broader distribution of operation duration in the conventional group.

Operation duration plays an essential role in the evaluation of surgical techniques. Longer operation duration may increase the risk of complications. Some investigators argued that ultrasonic surgery generally prolonged the operation time due to its lower efficacy during bone cutting^26^. This might be true because the ultrasonic surgical system seemed less effective in bone cutting than conventional rotary instruments. However, the average operation duration per joint showed no statistical significance in our study. This was because we recorded the operation duration from the skin incision to closure. Although the conventional method might be faster during bone cutting, it required more attention and time to archive adequate exposure and protect the soft tissue from potential injury. Moreover, we observed a significant positive association between blood loss and operation duration. We would predict an increase of 66.7 ml blood loss as the operation duration was lengthened by 1 hour, holding other variables constant. The operation duration had the most significant effect on blood loss (0.72), following by technique (0.39). Intergroup comparison of the linear models further confirmed that there was a similar positive linear association between blood loss and operation duration regardless to the technique, except the broader distribution of operation duration in the conventional group **(**Fig. 1**)**. To further detect if there was multicollinearity between predictor variables, we calculated variance inflation factor (VIF). Results indicated no significant multicollinearity existed (VIF = 1.09 for technique, VIF = 1.08 for operation duration, mean VIF = 1.11 for all variables).

**Figure 2:**
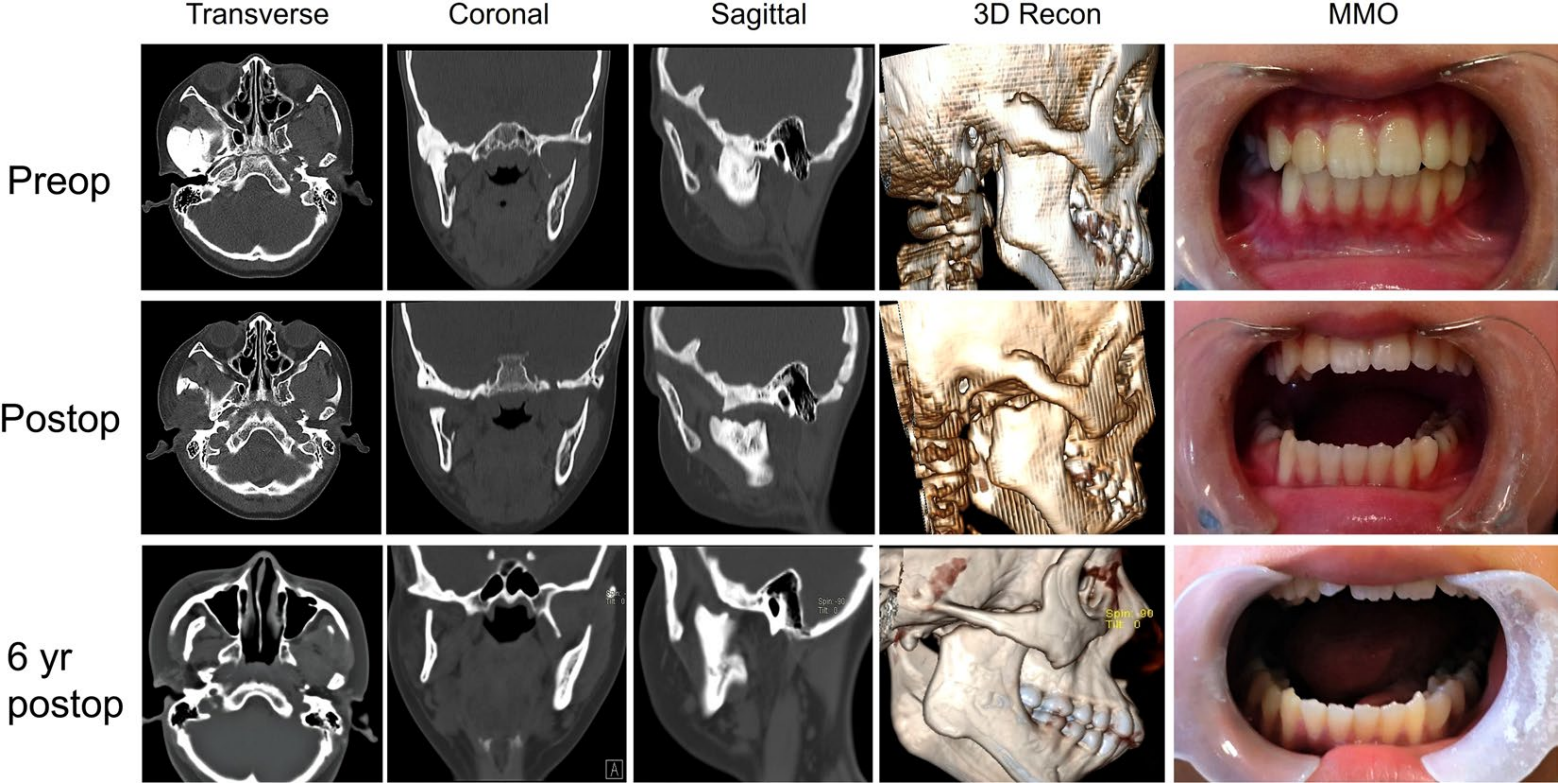
Preoperative, postoperative and 6 years postoperative follow-up CT scan and MMO showed a substantial reduction of the ankylotic block and stable improvement of MMO. (**a**) Preoperative transverse CT scan; (**b**) preoperative coronal CT scan; (**c**) preoperative sagittal CT scan; (**d**) preoperative 3D reconstruction of CT scans; (**e**) preoperative MMO; (**f**) postoperative transverse CT scan; (**g**) postoperative coronal CT scan; (**h**) postoperative preoperative sagittal CT scan; (**i**) postoperative 3D reconstruction of CT scans; (**j**) postoperative MMO; (**k**) 6 years postoperative transverse CT scan; (**l**) 6 years postoperative coronal CT scan; (**m**) 6 years postoperative sagittal CT scan; (**n**) 6 years postoperative 3D reconstruction of CT scans; (**o**) 6 years postoperative MMO.

Patients with ATMJ present with a progressive limitation of mouth opening. Present study showed an average preoperative MMO of 4.3 ± 4.1 mm, whereas normal MMO was approximately 50 mm. This resulted in a serious impairment of oral function including mastication, swallowing, breathing, and speech. To regain a functional MMO and avoid relapse, extensive resection of the bone mass is of great importance during the surgery. A minimum 15 mm gap between the mandibular ramus and glenoid fossa, as well as an intraoperative passive MMO greater than 35 mm, was recommended^27^. This required an adequate resection of the ankylotic block which can be achieved by using both ultrasonic and conventional bone cutting techniques. We found no significant differences in preoperative, intraoperative and a minimum 2-year postoperative MMO between two groups, indicating both techniques were effective in treating ATMJ and maintaining long-term effectiveness. In the ultrasonic group, the average improvement of MMO (31.44 ± 5.78 mm) was substantial, and the relapse of MMO (1.6 ± 2.9 mm) was insignificant. This was further confirmed by the follow-up CT scanning and three-dimension reconstruction (Fig. 2). However, we noticed that four patients (3 patients with type IV ATMJ, 1 patient with type III ATMJ), two in each group, experienced malocclusion and mandibular deviation to the surgical side during opening mouth 2 to 5 years postoperatively.

The heat produced during the ultrasonic bone cutting was less than conventional rotary and reciprocating instruments. Previous studies showed higher osteoblast viability and undamaged collagen around the cutting margins of ultrasonic osteotomy due to the lower temperature, whereas conventional osteotomy resulted in thermal damage and micro-osteonecrosis^28,29^. Gulnahar *et al*.^30^ found that the expression of heat shock protein 70, an indicator of thermal damage, trauma, and inflammation, was twice higher in conventional than ultrasonic surgery. Furthermore, faster osteogenesis with greater mineralization and vascularization was observed in the ultrasonic group compared to the conventional group^31^.

A major disadvantage of the ultrasonic surgery was the cost of using this system and the expensive consumables^32^. However, this flaw cannot obscure many abovementioned virtues. Limitations of this study included a small sample size. We might be able to detect the statistical significance of more outcomes given more participants. Other inherent limitations as an observational study also existed such as unrealistic to make causal inference due to possible confounders and bias. Randomized controlled trial with adequate sample size is needed to elaborate a causality in the future.

In conclusion, our study showed that gap arthroplasty using ultrasonic bone cutting technique was associated with less intraoperative blood loss and stable long-term improvement of MMO when compared to conventional osteotomy for the treatment of ATMJ.

## Materials and Methods

### Participants

After the approval of the Medical Ethical Committee of Chinese PLA General Hospital, we designed a prospective cohort study on 25 consecutive patients who presented to Department of Oral and Maxillofacial Surgery and Department of Plastic Surgery, Chinese PLA General Hospital from March 01, 2012 to March 01, 2016. Informed consent was obtained from all participants or their legal guardians. All methods were performed according to the relevant guidelines and regulations. Patients were followed up for at least 2 years. Follow-up visits were scheduled at 1 week, 1 month, 6 months, and then annually after surgery at Chinese PLA General Hospital. Data was collected by the same group of investigators (TJ, LW, RZ, LZ, LX) from the electronic medical record system.

Inclusion criteria were: (1) Type II, III or IV ATMJ classified according to Sawhney’s classification^33^ as follows. Type II: Consolidation of the deformed head of the condyle, articular surface mainly at the post edges and the middle part uninvolved; Type III: Ankylotic mass involves the mandibular ramus and zygomatic arch; Type IV: TMJ is replaced entirely by heterotopic bone. The type of ATMJ was diagnosed by clinical and radiological assessment; (2) ATMJ was released with gap arthroplasty; (3) Participants underwent removal of the ankylotic mass using either ultrasonic surgical system or conventional rotary and reciprocating instruments. Exclusion criteria are: (1) Type I ATMJ; (2) Patients received interpositional or reconstructive arthroplasty; (3) Patients had previous TMJ surgery; (4) Incomplete follow-up data.

### Procedures

**Figure 3:**
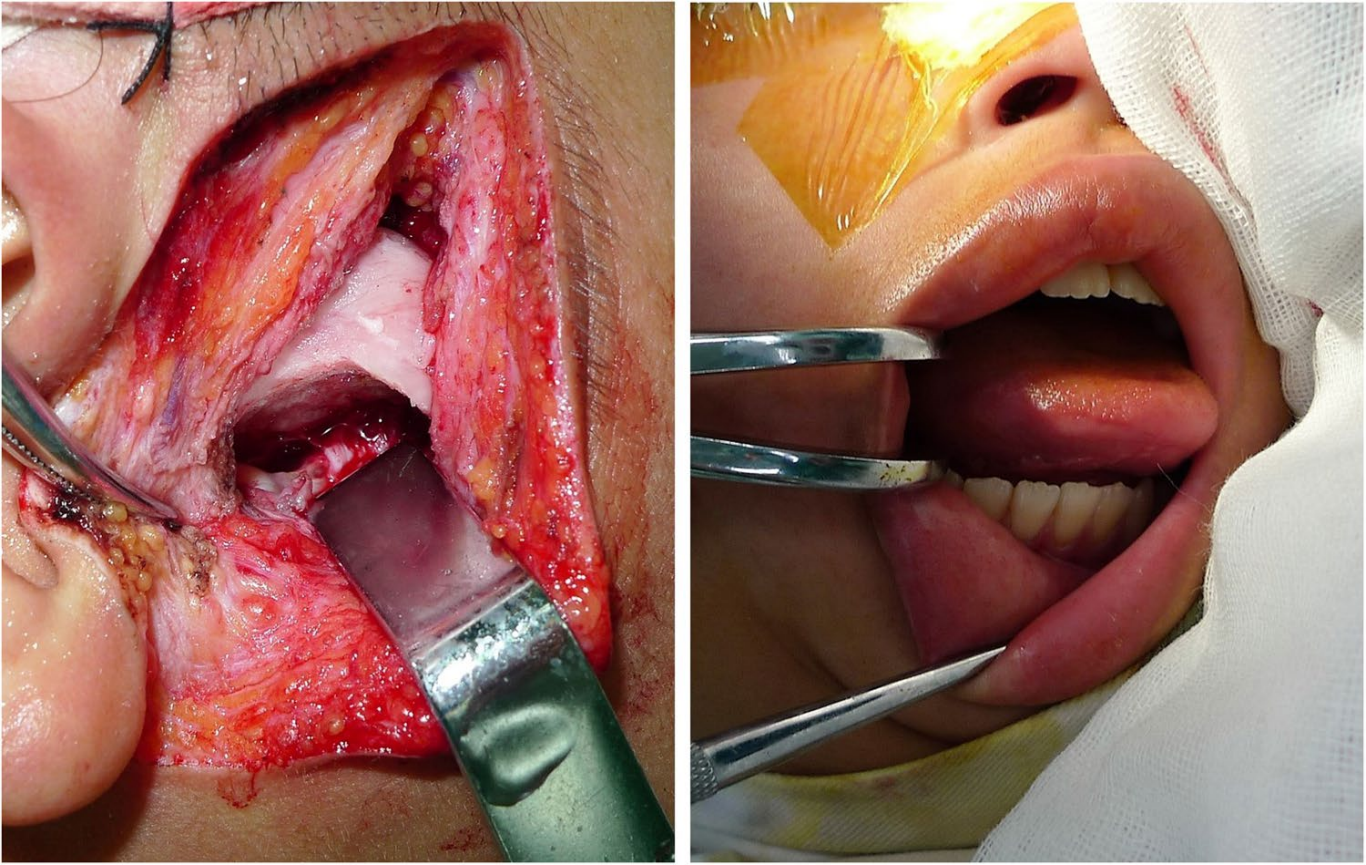
(**a**)A ≥ 15 mm gap between the ramus and the glenoid fossa with (**b**) a minimum mouth opening of 35 mm was achieved after removal of the ankylotic mass.

All surgeries were performed by the same group of senior surgeons under general anesthesia with nasotracheal intubation according to previous studies^34–36^. Surgical procedures and perioperative cares in the ultrasonic group such as surgical approach (preauricular approach with temporal extension if needed), exposure, hemostasis, and wound closure were in accordance with those in the conventional group. The ultrasound generator (SonicMed, Beijing, China) was set as 16 W output and 30–50 kHz ultrasonic frequency. After removal of the ankylotic mass, a minimum of 15 mm gap between the ramus and the glenoid fossa **(**Fig. 3a**)** with a minimum mouth opening of 35 mm **(**Fig. 3b) was achieved. Incisions were closed in layers. All patients started physiotherapy 10 days postoperatively.

### Variables

Predictor variables included patient age, sex, number, type, and etiology of ATMJ, technique used, preoperative MMO, and operation duration per joint. The etiology of ATMJ was classified into trauma, infection, rheumatic disease, previous surgery, and other. The operation duration per joint was recorded from skin incision to closure. The primary outcome variable was the intraoperative blood loss per joint, which consisted of two parts: (1) The blood suctioned by the vacuum suction system; (2) The blood absorbed by the surgical gauzes, which was calculated by the weight of used gauzes minus the weight of preoperative gauzes. The volume of absorbed blood = the weight of absorbed blood/1.05. Therefore, the total volume of blood loss = the volume of fluid in the vacuum suction jar + the volume of absorbed blood - the volume of irrigation sterile saline solution. The secondary outcome was the long-term improvement of MMO. MMO was defined as the interincisal distance when the mouth was opened at the maximum extent and measured by using a caliber at 3 time points: preoperative (t0), intraoperative(t1) and a minimum 2-year postoperative (t2). Long-term improvement of MMO was calculated by MMO(t2)-MMO(t0). Relapse of MMO was defined as MMO(t2)-MMO(t1). If the MMO(t2) was greater or equal to MMO(t1), relapse was considered as none. Reankylosis was defined as a less than 25 mm of MMO(t2). Other outcomes included drainage duration, hematoma, infection, facial nerve injury, and pain. Drainage was stopped when the color of fluid became lighter and daily volume of fluid was less than 5 ml. Hematoma was defined as a localized swelling filled with clotted blood. Infection was defined as redness, swelling, pain, and possible concomitant purulence at a surgical site within 14 days after surgery. A positive microbiological culture was required to confirm the infection and pathogens. Facial nerve injury was evaluated by using the House-Brackmann facial nerve grading system^37^. Pain was assessed within 7 days postoperatively by using a visual analogue scale, 0 as no pain and 10 as unbearable pain^38^.

### Statistics

Continuous variables were expressed as means ± standard deviations if they were normally distributed. Categorical variables were reported as frequencies or proportions. The differences of variables between the two groups were compared with Student’s t-test for continuous data and Fisher’s exact test for categorical data. Pearson correlation analysis was performed among age, sex, number, type, etiology of ATMJ, technique used, preoperative MMO, operation duration, and blood loss. Univariate linear regressions were performed between the blood loss and each predictor variable. Since we have a small number of observations, LASSO regression was performed for variable selection. Multivariate linear regression including age, sex, type of TMJ, technique used, preoperative MMO and operation duration was performed. A two-sided p-value less than 0.05 was considered statistically significant. Statistical analysis was performed using STATA v15.0 (Stata Corporation, College Station, TX, USA).
